# Reasons why Thai patients with chronic kidney disease use or do not use herbal and dietary supplements

**DOI:** 10.1186/1472-6882-14-473

**Published:** 2014-12-06

**Authors:** Mayuree Tangkiatkumjai, Helen Boardman, Kearkiat Praditpornsilpa, Dawn-Marie Walker

**Affiliations:** Division of Primary Care, School of Medicine, University of Nottingham, QMC, Nottingham, NG7 2UH UK; Division of Social Research in Medicines and Health, School of Pharmacy, University of Nottingham, Nottingham, UK; Division of Nephrology, Department of Medicine, Faculty of Medicine, Chulalongkorn University, Bangkok, Thailand

**Keywords:** Herbal medicine, Dietary supplements, Reason, Chronic kidney disease, Thailand

## Abstract

**Background:**

Despite a high prevalence of herbal and dietary supplement use (HDS) in pre-dialysis patients, the reasons are unknown as to why they decide to use HDS. Objectives of the cross-sectional and qualitative studies were to determine reasons for the use and non-use of HDS in Thai patients with chronic kidney disease (CKD).

**Methods:**

This prospective study recruited 421 patients with stage 3–5 CKD from two kidney clinics in Thailand, and 357 were followed up regarding their HDS use over 12 months. Patients receiving renal replacement therapy at baseline were excluded. Participants were interviewed at baseline and in the twelfth month regarding their HDS use, and reasons for their use or non-use of HDS. Among HDS users, 16 patients were enrolled in a qualitative study and were interviewed using eight-open ended questions about reasons for HDS use. Descriptive and thematic analyses were performed.

**Results:**

Thirty-four percent of patients with CKD consistently used HDS over the 12 months and 17% of all patients intermittently took them during the follow-up period. At baseline, family or friends’ recommendation was the most common reason for HDS use (35%), followed by having a perception of benefits from using HDS (24%). During the follow-up period, perceived benefits of HDS was a frequently reported reason for either continuing with HDS use (85%) or starting to use HDS (65%). Negative experience from using HDS influenced patients to stop using them (19%). Although the main reason for non-use of HDS was trust in a doctor or effectiveness of conventional medicine (32%), doubt about the benefits from HDS or concerns about negative effects were frequently reported reasons for non-use (23%). Doctor’s recommendations to avoid using HDS were the main influence for non-users (19%) and for those who had stopped using HDS (23%). The media and patients’ social network had an impact on HDS use.

**Conclusions:**

Patients who perceived benefits from HDS use were more likely to use HDS, whilst non-users had negative attitudes towards HDS. Health professionals therefore should educate patients and their relatives about the risks and benefits from using HDS.

## Background

Use of herbal and dietary supplements (HDS) has risen worldwide [1]. Patients with kidney diseases, such as chronic kidney disease, dialysis, and kidney transplantation, are more likely to use HDS (28%-57%) [2–5]. Most frequently reported reasons for using HDS or complementary and alternative medicine (CAM) in patient populations are the perception of their benefits and safety, dissatisfaction with conventional medicine, and willingness to try them [6–17]. Cross-sectional studies are the main information source reporting this issue, whilst there are limited qualitative studies [18–20]. Small numbers of surveys report reasons why patients are unlikely to use HDS or CAM, that is, doubt about their efficacy and safety, and satisfaction with conventional medicine [15, 21–23].

There are few surveys about reasons for HDS or CAM use amongst patients with kidney diseases [2, 4, 24]. They report that the perceived benefits and safety of HDS or CAM are the main reasons for their use. Shah et al. (2013) found CAM use was related to experiencing side effects from conventional medicine (OR 9.59, 95% CI 2.77-33.20), dissatisfaction with a doctor (OR 4.16, 95% CI 1.75-10.10) and belief in a holistic approach (OR 3.20, 95% CI 1.58-6.47) [24]. However, there is a lack of qualitative studies conducted exploring this issue further. Therefore in this paper, a combination of a survey and qualitative study aimed to ascertain reasons why patients with chronic kidney disease (CKD) use HDS. The secondary objective of the survey was to determine reasons for non-use of HDS. The patients were also followed up over 12 months in order to observe decision-making process in HDS use or non-use. These findings will guide health professionals into fully understanding of HDS use in these patients and prepare them to deal with this issue.

## Methods

### The survey

A prospective, cross-sectional study recruited 421 patients with stage 3–5 CKD from two kidney clinics at teaching hospitals in Thailand during January to June 2012. All patients, in both settings, were approached by MT or two trained interviewers, and informed about the project, and asked for their consent. Patients receiving renal replacement therapy before the recruitment were excluded. Fifteen patients did not meet the inclusion criteria due to three patients receiving dialysis and 12 patients having stage 2 CKD (estimated glomerular filtration rate of approximately 60–65 ml/min/1.73 m^2^). Of 406 patients recruited, 357 patients were followed up regarding their HDS use, and any perceived beneficial and detrimental effects of HDS over 12 months. Forty-nine patients left the study due to death (n = 30) or lost to follow-up (n = 19). Herbal and dietary supplements were defined as products containing plant-derived material, either raw or processed ingredients, from one or more plants, or containing dietary ingredients, such as vitamins, minerals, amino acids and substances, such as, enzymes, organ tissues, glands and metabolites [25, 26]. Ethical approval was obtained from the Institutional Review Board for Research in Human Subjects at Faculty of Medicine, Chulalongkorn University and Srinakharinwirot University in Thailand, and the Medical School Research Ethics Committee, University of Nottingham in the UK.

Patients were interviewed at baseline regarding their HDS use, information sources, and reasons for HDS use or non-use. The data collection was via a researcher-administered questionnaire, which was developed and validated by the authors [27]. The questionnaire used a mixture of previously validated and new questions. In the twelfth month, participants were interviewed over the telephone about their HDS use and reasons for continuing, stopping or starting HDS use since the baseline survey. Simple frequencies with percentages were used to determine reasons for HDS use and non-use.

### The qualitative study

From the HDS users identified in the survey, the patients who had provided details of the reasons for HDS use were approached. If they consented to participate in the qualitative study, they were recruited. Patients were recruited and interviewed face-to-face until data saturation was reached (n = 16). Eight open-ended questions about their reasons for HDS use were developed based on literature, see Table 1 [28, 29]. This interview was piloted and it was found that all participants (n = 6) understood and answered the questions fully. This questionnaire was administered using face-to-face interviews. The interviews lasted approximately 5–10 minutes and were audio recorded.

**Table 1:** **The interview questions about reasons for HDS use**

	Eight-item interview questions
1.	How and when were you introduced to HDS?
2.	Why do you use HDS?
3.	What led you to start using HDS?
4.	Did anything influence you to start using HDS, e.g. advice from friends, doctor, news reports, etc.?
5.	Are there benefits of HDS compared with conventional medicines? Please explain why you think this.
6.	What did you hope taking HDS would achieve?
7.	Do you have any concerns about using HDS? If yes, what? Then please compare with conventional medicines.
8.	Have you had any warnings about taking the HDS such as from doctors, friends? If yes, has it influenced your use in anyway?

The audio recordings were transcribed verbatim and the Thai transcripts were twice checked for accuracy against the recordings before starting the process of forward translation. Meaning-based translation from Thai language to English language was performed and English transcripts were twice checked with the Thai transcripts [30]. Then five out of the sixteen transcripts were backward translated by a bilingual person which found one error. This was rectified.

The transcripts were analysed by inductive thematic analysis with line-by-line coding [31, 32]. The Weft QDA, a software programme for qualitative data analysis, was used for assisting in the organisation of the transcripts [31, 32].

## Results

Demographic characteristics of participants are shown in Table 2. Of 357 participants, 166 participants (46%) had used HDS together with conventional medicines in the last 12 months before the recruitment. Three patterns of HDS use were seen over the year: continuing users (34%, n = 123), non-users (49%, n = 177) and intermittent users (17%, n = 60).

**Table 2:** **Demographic characteristics of respondents in the survey (n = 357) and qualitative study (n = 16)**

Demographics	The survey (n = 357)	The qualitative study (n = 16)
	Frequency (Percentage)	
Mean age and SD	66 ± 13 years	62.5 ± 12.3 years
Sex		
Male	162 (45.4)	6 (37.5)
Female	195 (54.6)	10 (62.5)
Current address		
Bangkok	132 (37.0)	6 (37.5)
Rural areas	225 (63.0)	10 (62.5)
Education		
Primary or secondary school	254 (71.1)	8 (50.0)
Higher education	103 (28.9)	8 (50.0)

**Figure 1:**
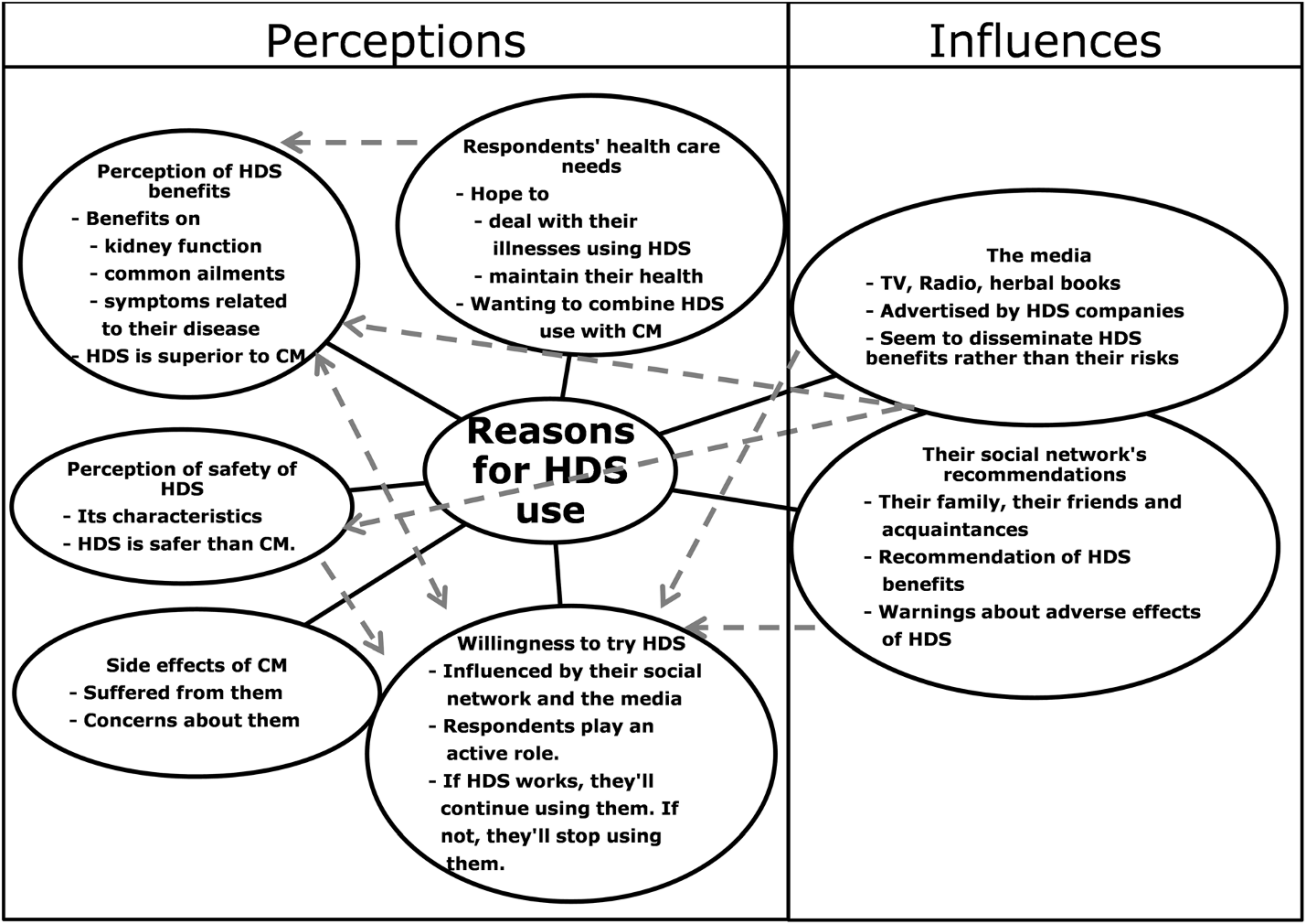
**Themes from interviews with 16 participants about their reasons for using HDS.**

The most frequently reported reasons for HDS use in the survey were due to family or friends’ recommendations (35%), followed by the expectation of gaining benefit from using HDS (24%), see Table 3. Meanwhile, only 3% of users reported the safety of HDS as a reason for HDS use. Regarding the qualitative findings, five themes emerged on perceptions of HDS: patients’ health care needs; perceptions of benefit and safety of HDS; willingness to try HDS; and side effects of conventional medicine (CM). Additionally, two further themes influencing HDS use were recommendations from patients’ social network and the media, see Figure 1. Such influences were consistent with the findings from the survey; family, friends and the media were most frequently reported as HDS information sources, see Table 3. The main findings of the survey and qualitative study were similar, except that health professionals’ recommendation for HDS use and the ease of access to HDS were not found in the qualitative study.In the qualitative study, the media and the patients’ social network influenced patients to try HDS as they provided information about the benefits and safety of HDS (Figure 1). Participants also seemed to respect educated family members, friends and acquaintances, such as doctors, nurses, and teachers, so they complied with their recommendation to use HDS. Both the media and the social network provided limited information about the safety of HDS use in patients with CKD.

**Table 3:** **Reasons for HDS use from both quantitative and qualitative studies at baseline**

Quantitative results (n = 166)		Qualitative results (n = 16)
Question	Frequency (%)	
Reasons why HDS used (n = 271)*		
Family/friend’s recommendation	95 (35)	Influenced by their social network who were health care professionals or teachers (n = 9)
HDS will work	65 (24)	Perception of their benefits
Willing to try anything that helps	53 (20)	(n = 11)
Prefer to use HDS	26 (10)	Health care needs (n = 7)
		Willing to try (n = 4)
		Intention to use (n = 2)
Health care provider’s recommendation	17 (6)	No mention
Safer than CM or no adverse effects from using HDS, compared with CM	8 (3)	Perception of their safety (n = 5)
		Their characteristics
		No or little side effects
		Safer than CM
		Had experiences or concerns about adverse effects of CM (n = 2)
Easy access	4 (2)	No mention
Recommended by traditional practitioners or HDS sellers	2 (< 1)	Their family recommended and then consulting Chinese herbal medicine practitioners (n = 1)
Recommended by fellow patients	1 (< 1)	Influenced by their social network (n = 9)
Information sources (n = 188)*		
Family and friends	100 (53)	Influenced by their social network (n = 9)
TV, radio, internet, leaflets, books or scientific evidence	59 (31)	Influenced by the media (n = 9)
Practitioners	18 (10)	No mention
HDS sellers	6 (3)	No mention
Own knowledge of HDS	4 (2)	No mention
Another patient with CKD	1 (1)	No mention

*Participants were able to report more than one reason or information source, so these total more than 166.

CM = Conventional medicine.

*… a herbal company advertises on television that the product is approved by the Thai FDA for a dietary supplement… A herbal company advertises “This herb is the best on sale product” “It is useful” “You would not be disappointed”. (R13, m age 70)*

*My younger brother confirmed that HDS is good and cleans the blood vessels, so blood circulation is improved. He said “You should take it” “It does no harm”. (R4, f age 56)*

*He [my older brother who has a wife working as a nurse and whose brother-in-law is a doctor] said “You should eat these herbs, they are good”. (R13, m age 70)*

*You know, there is marked lack of warning about taking herbs. (R11, f age 44)*

However, some patients were concerned about renal adverse effects of HDS, so they tended to use HDS with caution and would consult with their health care providers if necessary.

*If I take too many herbs, I’m afraid of worsening liver and kidney function. (R7, m age 67)*

Patients’ desire to improve, or at least stabilise their condition was a drive for seeking alternative therapy, for example avoidance of receiving dialysis and being able to live their normal daily lives, such as being able to walk and cook. As a result, some patients actively sought HDS information, particularly about their benefits, and decided to use HDS by themselves. They experimented with HDS and monitored their effects. Where they noticed positive effects from using HDS, they would continue using them and where they did not, they would stop using them. Others wanted to integrate HDS into their mainstream treatment in order to increase the efficacy of the conventional treatment.

*I’m afraid of receiving dialysis… I want to use everything, which helps me to avoid receiving dialysis. (R8, m age 67)*

*I have to learn about herbal information by myself and know about them from my colleagues’ or friends’ experiences of using herbs… I have to think whether or not herbs suit me and decide to use them by myself. (R11, f age 44)*

*I wanted to try them. After trying them, they were good. Thus, I continue to use them. (R1, f age 59)*

*Conventional medicines are more effective than any herbs. Herbs supplement conventional medicines. (R12, m age 75)*

Some patients reported using HDS due to dissatisfaction with conventional medicine or had negative attitudes towards it.

*I had lots of side effects from conventional medicines, so I turned my thoughts to herbal use and used it. (R5, m age 43)*

*I’m afraid of worsening kidney function. If I take lots of prescribed medicines, whether they will affect kidneys or not? (R9, f age 46)*

Regarding the follow-up study, reasons for continuing or changing are shown in Table 4. The perception of benefits from using HDS was the main reason for consistent users (85%, n = 105) and new users (65%, n = 11). Amongst 73 patients who continued using HDS due to the benefits gained from using HDS, 31 patients (42%) reported that they relieved minor ailments, such as kariyat or vitamin C for the common cold, curcumin for flatulence, senna or ‘Ka Sai’ as a laxative, and ‘Ya Hom’ for dizziness. Twenty-five patients (34%) had used dietary supplements for well-being, and perceived such effects during the follow-up period. Examples of such products were ginseng, essence of chicken drink, fish oil, bee pollen, germ oil, swiftlet’s nest drink, and botanical extracts. Other benefits from using HDS are shown in Table 5. Neither negative experiences from HDS use (26%, n = 11) nor negative attitudes towards HDS (7%, n = 3) influenced continuing HDS use. Eight patients experienced negative effects from using HDS, see Table 5. Three patients perceived no benefit from using HDS, i.e. essence of chicken drink for tiredness, seeds of *Moringa oleifera* for treatment of their chronic illnesses, and mixed vegetable and fruit beverage for diabetes, see Table 4.

**Table 4:** **Reasons why patients decided to use or stop using HDS at the end point (n = 357)**

Reasons	Frequency (%)
Continuing to use HDS (n = 123)	
Gaining benefit from using HDS	73 (59)
Expecting to gain benefit	32 (26)
To supplement their diet	8 (6)
Their family members or friends provided HDS	6 (5)
Recommended by their doctor	2 (2)
Used to take it	1 (1)
HDS is safe	1 (1)
Stopping HDS use (n = 43)	
Recommended by their doctor	10 (23)
Having adverse effects from using HDS	8 (19)
Their minor ailments relieved	8 (19)
Do not want to use	5 (11)
Experience with no benefit from using HDS	3 (7)
Concern about adverse effects on kidneys	3 (7)
Cannot afford or not available	3 (7)
Receiving dialysis or their disease worsened	2 (5)
Taking high numbers of conventional medicines	1 (2)
Starting to use HDS (n = 17)	
Expecting to gain benefit	11 (65)
Their family members or friends provided HDS	3 (18)
Their disease worsened	1 (6)
No reason given	2 (12)

**Table 5:** **Herbal and dietary supplements and respondents’ experiences on benefits or adverse effects**

Types of HDS (n = 14)	Experience on benefits from using HDS during the follow-up period
Three different types of mushrooms, jujube (*Zizyphus mauritiana*) and roselle (*Hibiscus sabdariffa*), and *Boesenbergia rotunda*	Stable serum creatinine
A Chinese combination: Cordyceps, *Angelica sinesis*, deer antler velvet, five flavour berry (*Schisandra chinensis*) and cinnamon	Stable serum creatinine
A herbal combination: *Boesenbergia rotunda*, mint, ginger, galangal, lemongrass, kaffir lime leaves and shallots	Stable serum creatinine
*Boesenbergia rotunda*	Stable serum creatinine
*Boesenbergia rotunda* and Chinese herbal medicine	Stable serum creatinine
*Moringa oleifera*	Stable serum creatinine
Blue pea (*Clitoria ternatea*)	Stable serum creatinine
Spring bitter cucumber (*Momordica cochinchinensis*) and East Indian screw tree (*Helicteres isora*)	Delay in receiving dialysis therapy
Vap ca (*Houttuynia cordata*)	Diuretic effects
Lime (*Citrus aurantifolia*)	
*Clerodendrum petasites* (n = 2)	Diuretic effects
Bitter melon (*Momordica charantia*)	Decreased blood sugar
*Centella asiatica* and *Moringa oleifera*	Decreased blood pressure
**Types of HDS (n = 8)**	**Experience on adverse effects from using HDS during the follow-up period**
Protein supplements	Proteinuria
Wheatgrass	Tinnitus
Essence of chicken drink	Increased blood sugar
Germ oil	Increased body weight
River spiderwort (*Tradescantia fluminensis*)	Increased serum creatinine
Unknown Thai traditional medicine	Nausea
Thai herbal remedy containing aloe for laxative	Increased serum creatinine
Thai traditional medicine for cancer called ‘Luke Klon’	Increased serum creatinine

Three unknown Thai or Chinese herbal remedies were reported on a benefit of stable serum creatinine.

Two patients were recommended by their doctor to use fish oil for the prevention of cardiovascular disease, vitamin E for CKD and vitamin C for the common cold. Twenty-three percent (n = 10) of those who stopped were advised by their doctor to avoid using HDS, i.e. Thai traditional herbal remedies, *Moringa oleifera*, rice bran oil, Chinese traditional medicine, curcumin, germ oil and chlorophyll.

Reasons for non-use of HDS at baseline are shown in Table 6. The most frequently cited such reasons were either trust in their doctor or a belief in the benefits from conventional medicine (32%, n = 62), followed by health care professionals’ recommendations to avoid HDS (19%, n = 36). Eight participants (5%) reported that they had no particular reasons for choosing not to use HDS.

**Table 6:** **Reasons for not using HDS at baseline (n = 194)** ^**a**^

Reasons	Frequency (%)
Patients trusted their doctor or trusted/needed to use conventional medicines or perceived benefits of conventional medicines are superior to HDS	62 (32)
Health care providers^b^ advised that the patient should not use HDS	36 (19)
Experiences or concerns about harm from HDS^c^	30 (16)
Doubt about benefits of HDS or experience with no benefits from using HDS	14 (7)
Don’t want to use HDS	13 (7)
Taking a high number of conventional medicines	10 (5)
Had renal insufficiency, so patients concerned about harm from HDS	9 (5)
HDS are expensive or HDS are not available in their area	6 (3)
Patient’s relatives recommended that they should not use HDS	5 (3)
Don’t know enough information about HDS^d^	4 (2)
They perceived that they are well	3 (2)
A book about kidney diseases indicated that CKD patients should not use HDS	1 (1)
A patient need not use HDS if (s) he adheres to medication and dietary recommendations for CKD patients	1 (1)

^a^Participants were able to report more than one reason, so these total more than 191; Missing data was 15 participants (7.8%).

^b^Doctors or Pharmacists.

^c^Adverse effects of HDS, contaminated HDS, or HDS-conventional medicine interactions.

^d^Information about indications, doses, benefits, or risks of HDS.

## Discussion

Over the 12 months of the study, most patients continued to use or not use HDS. It is clear that having either an expectation or perception of gaining benefit from using HDS was the most important factor influencing patients with CKD to start or continue using HDS in the present study. These findings are supported by a large number of other surveys in patients with chronic illnesses, including kidney diseases [4, 8, 14, 15]. Several studies in the US, Netherlands and Thailand also report that patients complement their conventional medicine with HDS similar to the findings in the present study [7, 10, 12, 13]. Some patients reported that they wanted to use HDS combined with their conventional medicine for incremental beneficial effects. This suggests that health care professionals and researchers should investigate how to integrate conventional medicine and HDS in order to complement their benefits and avoid detrimental effects in patients with CKD.

Meanwhile, patients who decided to stop, or not use, HDS was largely due to either experiences of adverse effects, a perception of no benefit from using HDS, or having negative attitudes towards HDS. This is consistent with a European survey in cancer patients [21]. In addition, health care professionals’ recommendation for avoiding HDS was a major influence on those who stopped or did not use HDS in the present study. This indicates that health care providers are likely to be a key decision-maker for the group. Further studies need to be investigated regarding the effect of the doctor-patient relationship on patients with CKD and their decision to use HDS or not.

In the current study, patients seemed to be motivated to use HDS as a last resort when they were not satisfied with what conventional medicine could achieve, such as a desire to avoid dialysis therapy [6, 14, 19]. Benefit and safety information about HDS were mainly provided by the media and patients’ family members or friends in the present study. Family members and friends are an influencing factor in patient’s decision-making regarding HDS use in Asian populations. This is supported by other studies of CAM use amongst patients with chronic diseases in Asian countries [2, 7, 8, 22, 33, 34]. This could be a result of the close knit family culture in Asian countries. In comparison with studies in Western countries which found that patients with chronic illnesses used HDS if their health care providers suggested it [4, 10].

A systematic review has shown that the mass media report positive effects of CAM rather than their negative effects [35]. This is similar to our qualitative study where a patient complained about the lack of warning about HDS use in patients with renal insufficiency. Both health care providers and policy makers, particularly in Thailand, should be concerned about the potential impact, and therefore information about both the risks and benefits of using HDS should be widely available, particularly for patients who may be more susceptible to adverse effects from using HDS, such as those with CKD.

It appears that most patients in our study were independent in their decision-making about HDS use as only a small number of patients (6%) used it on the recommendation of their health care provider. Some participants actively searched for information about the benefits and safety of HDS and experimented with HDS by monitoring efficacy and adverse effects. This is likely to be a common process in the decision-making in patients who are interested in alternative therapy [29]. Health care professionals should acknowledge this, and support access to appropriate information for such patients together with monitoring for any effects resulting from HDS use.

Safety of HDS (3%) was seen as less influential on HDS use than their perceived benefit (24%) in our study. Several users were concerned about the potential negative effects of HDS on their kidneys, so they used HDS with caution, such as by reducing the dose of their HDS, or only occasionally using HDS. This differs from Spanner and Duncan’s study (2005) in Canada where they found at least 3 in 4 patients with CKD thought that dietary supplements caused no harm and improved their condition [4]. Likewise, people in the UK perceived that herbal medicine is safer than conventional medicine [36]. It would seem that Thai patients with CKD are more aware of detrimental effects resulting from HDS use and avoid such effects in their own way. Health care providers should be prepared to advise these patients regarding how to safely use HDS.

Amongst patients who reported gaining benefits from using HDS over the 12 months, three quarters perceived that HDS either relieved their minor ailments, such as the common cold, flatulence and constipation, or supplemented their diet. Also, respondents reported that their doctor recommended fish oil for the prevention of cardiovascular disease, vitamin E for CKD, and vitamin C for the common cold. These benefits have been supported by evidence although there is controversy about such beneficial effects from fish oil and vitamin C [37–40]. Vitamin E has been recommended as a vitamin supplement when patients with CKD stage 3 to 5 have a deficit in vitamin E [41].

Respondents perceived that several herbal medicines could maintain their kidney function; however, the majority of studies to prove this effect are in vitro. *Hibiscus sabdariffa*, cordyceps, *Zingiber officinale*, shallots, *Moringa oleifera*, *Centella asiatica* can inhibit angiotensin converting enzyme, which is related to a decrease in proteinuria, and then to slow the progression of CKD [42–45]. This mechanism also supports *Moringa oleifera* and *Centella asiatica* for antihypertensive effects reported by respondents. Moreover, there has been no scientific evidence to support some herbal medicines for maintaining kidney function, such as *Zizyphus mauritiana*, *Boesenbergia rotunda*, *Clitoria ternatea, Momordica cochinchinensis* and *Helicteres isora*. Therefore, further research, particularly clinical trials, needs to be conducted regarding this issue before any recommendations can be made.

Likewise, *Houttuynia cordata* and *Citrus aurantifolia* for diuretic effects have limited evidence in vitro [46, 47]. *Clerodendrum petasites* has no scientific evidence to support their diuretic effect. In contrast, a randomised, controlled trial with a small number of patients with diabetic type 2 revealed that *Momordica charantia* decreased glycated haemoglobin (A1C) greater than placebo [48]. This evidence is consistent with respondent’s report.

Almost all adverse effects from using HDS reported by respondents have no further evidence to support their statement. A respondent in the present study found that Thai herbal remedy containing aloe was related to worsening kidney function. This is consistent with a case report; *Cape aloe* causes acute kidney injury [49]. ‘Luke Klon’ is a dosage form of Thai herbal remedy and may be contaminated with steroids [50], so this product may induce kidney injury. Thus, health care providers and patients should be aware of such detrimental effects.

The findings in this survey should be generalised with caution due to only two hospitals being used as study sites, although the study sample matches the Thai general population in terms of gender, educational level, living in urban or rural areas, smoking and drinking status [51]. Mean age in the current study (66 years) was no different to other studies of patients with CKD in Taiwan, the UK and Italy (65–67 years) [52–54].

## Conclusions

Positive attitudes towards herbal and dietary supplements in patients with CKD were the main reasons for HDS use, particularly their perceived benefit. Meanwhile, negative attitudes towards HDS or negative experiences from using HDS, including their doctor’s influence, motivated patients not to use, or to stop, using HDS. The media and patients’ social network seem to positively influence patients to use HDS. Thus, health care providers and policy makers in Thailand should acknowledge this influence and provide high quality information about beneficial and detrimental effects of HDS use for these patients. Further studies are required to investigate the effect of the doctor-patient relationship on decision-making regarding the use of HDS.
